# Genes Responsive to Elevated CO_2_ Concentrations in Triploid White Poplar and Integrated Gene Network Analysis

**DOI:** 10.1371/journal.pone.0098300

**Published:** 2014-05-21

**Authors:** Juanjuan Liu, Jianguo Zhang, Caiyun He, Aiguo Duan

**Affiliations:** 1 State Key Laboratory of Tree Genetics and Breeding, Research Institute of Forestry, Chinese Academy of Forestry, Beijing, China; 2 Key Laboratory of Tree Breeding and Cultivation of the State Forestry Administration, Research Institute of Forestry, Chinese Academy of Forestry, Beijing, China; Institute of Genetics and Developmental Biology, Chinese Academy of Sciences, China

## Abstract

**Background:**

The atmospheric CO_2_ concentration increases every year. While the effects of elevated CO_2_ on plant growth, physiology and metabolism have been studied, there is now a pressing need to understand the molecular mechanisms of how plants will respond to future increases in CO_2_ concentration using genomic techniques.

**Principal Findings:**

Gene expression in triploid white poplar ((*Populus tomentosa ×P. bolleana*) ×*P. tomentosa*) leaves was investigated using the Affymetrix poplar genome gene chip, after three months of growth in controlled environment chambers under three CO_2_ concentrations. Our physiological findings showed the growth, assessed as stem diameter, was significantly increased, and the net photosynthetic rate was decreased in elevated CO_2_ concentrations. The concentrations of four major endogenous hormones appeared to actively promote plant development. Leaf tissues under elevated CO_2_ concentrations had 5,127 genes with different expression patterns in comparison to leaves under the ambient CO_2_ concentration. Among these, 8 genes were finally selected for further investigation by using randomized variance model corrective ANOVA analysis, dynamic gene expression profiling, gene network construction, and quantitative real-time PCR validation. Among the 8 genes in the network, aldehyde dehydrogenase and pyruvate kinase were situated in the core and had interconnections with other genes.

**Conclusions:**

Under elevated CO_2_ concentrations, 8 significantly changed key genes involved in metabolism and responding to stimulus of external environment were identified. These genes play crucial roles in the signal transduction network and show strong correlations with elevated CO_2_ exposure. This study provides several target genes, further investigation of which could provide an initial step for better understanding the molecular mechanisms of plant acclimation and evolution in future rising CO_2_ concentrations.

## Introduction

According to reports of the National Oceanic and Atmospheric Administration, the average annual concentration of CO_2_ in the atmosphere was 393.84 µmol·mol^−1^ in 2012. This concentration is increasing every year and by 2050 it is projected to surpass 550 µmol·mol^−1^ and reach 700 µmol·mol^−1^ by the end of 2100 [1]. Understanding how plants will respond to future elevated CO_2_ concentrations will help us comprehend how they are currently responding and how they may have adapted to the increase [2].

Although the impact of elevated CO_2_ on plant growth, physiology and metabolism has been extensively studied [1], [3], [4], the underlying molecular mechanisms of these changes are less understood. Some research has been done on these molecular mechanisms [5], [6], [7], but it is not yet very clear how gene expression varies in response to increased CO_2_ concentrations. In order to understand the molecular basis of the CO_2_ response, genomic and genetic tools such as microarray have been used in recent years [8], [9], [10], [11]. Among the plants studied, *Populus* is recognized as a model tree genus [12], [13], as it has many advantageous characteristics for genomic and genetic studies [14]. Therefore, in the present study, *Populus* was used for further analysis.

However, limited information is available at the transcriptome level in *Populus* under elevated CO_2_, and such information may allow us to understand plant adaptation and evolution as CO_2_ rises [15]. Recent studies using cDNA microarrays and transcriptome analysis revealed gene expression changes during senescence caused by elevated CO_2_ (550 µmol·mol^−1^) in *P.*× *euramericana* [13]. Gene expression in leaves is sensitive to the elevated CO_2_ (550 µmol·mol^−1^), depending on the developmental leaf age in *P.*× *euramericana* [11]. Comparing the leaf transcription profiles, different genotypes of *P. tremuloides* show significant variation in gene expression when exposed to CO_2_ elevated to 560 µmol·mol^−1^ [16]. The expression of 4600 expressed sequence tags in poplar were investigated by Gupta et al. [17], who first reported the gene expression in response to elevated CO_2_ (560 µmol·mol^−1^) and/or O_3_ in *P. tremuloides*. The first comprehensive analysis of gene expression in leaf and stem of *P. deltoides* under higher CO_2_ concentrations (800 and 1200 µmol·mol^−1^) was reported by Druart et al. [18]. However, earlier studies focused on CO_2_ concentrations of ∼550 µmol·mol^−1^. Here, we designed experiments with exposure to the current and two future atmospheric CO_2_ concentrations (550 and 720 µmol·mol^−1^) and used microarray analysis to delineate their effects in terms of the underlying molecular network in order to test the hypothesis that gene expression in leaves changes under these conditions.

Given this aim, it is necessary to use bioinformatics methods to understanding crucial factors that control the leaf gene expression affected by elevated CO_2_. In turn, an integrative analysis that combines changes in gene expression with gene functions within a genetic network helps us elucidate the molecular mechanisms with elevated CO_2_ exposure. Furthermore, we present the first integrated gene network analysis to identify several key genes that are most associated with elevated CO_2_ treatments in a polyploidy plant.

## Materials and Methods

### Plant material and experimental treatments

On 11 March 2010, homogeneous 20 cm long, woody-stem cuttings of triploid white poplar ((*P. tomentosa ×P. bolleana*) ×*P. tomentosa*) were planted in 20 cm×26 cm×34 cm plastic pots with a mixture of clay soil/sand/peat moss (5∶3∶2). Twenty randomly selected pots were moved into three controlled environment chambers (AGC-2, Zhejiang University Electrical Equipment Factory, Hangzhou, China) on 15 June 2010. Each chamber measured 3.5 m×2.2 m×3.2 m (L×W×H), with a relative humidity of 65±5%, and an average daytime photosynthetic active radiation of 800 µmol·m^−2^·s^−1^. They were exposed to different CO_2_ concentrations for three months from 25 June 2010.

The three CO_2_ concentration treatments were: T0 treatment, 385 µmol·mol^−1^ CO_2_, day 25°C/night 20°C; T1 treatment, 550 µmol·mol^−1^ CO_2_, day 28°C/night 23°C; and T2 treatment, 720 µmol·mol^−1^ CO_2_, day 31°C/night 26°C. The concentrations of CO_2_ were continuously and strictly monitored by an automatic CO_2_ detection system in each AGC-2 chamber. In each chamber, the pots were rotated once per week to minimize the effects of microclimatic variation within the chambers.

### Physiological measurements and ELISA

The height and stem diameter of twenty plants from each chamber were measured on the first day (Jun 25) and after 3 months (Sep 25) under each CO_2_ concentration. Tree height was measured from the base of the main stem to its apex, and diameter was measured at the base of the main stem. At the same time, ten trees were randomly selected for determination of the maximum net photosynthesis rate. The measurements were made from three fully expanded leaves in the middle portion of each stem, using a portable photosynthesis system (LI-6400; Li-Cor, Lincoln, NE, USA).

The extraction, purification and quantification of the endogenous phytohormones indole-3-acetic acid (IAA), gibberellic acid (GA_3_), abscisic acid (ABA) and cytokinin zeatin riboside (ZR), was performed according to Wang et al. [19]. ELISA kits (Chemical Control Technology Laboratory, Beijing, China) used for estimation of the hormone levels came from China Agricultural University (Beijing, China).

Each measurement was repeated three times. One-way analysis of variance (ANOVA) and least significant difference (LSD) test were used to determine significant differences in growth, net photosynthesis rate, and hormone content. Statistical significant was set as *P* = 0.05.

### RNA isolation and quality assessment

After three months of continuous treatment, healthy leaves from three individual plants in each chamber were harvested after physiological measurements. Samples were immediately frozen in liquid nitrogen and stored at −80°C. Total RNA was extracted and purified using RNAqueous phenol-free total RNA isolation (Ambion, Austin, TX, USA) and Plant RNA Isolation Aid (Ambion) following the manufacturer's instructions and checked for RNA integrity number to assess the RNA integration with an Agilent Bioanalyzer 2100 (Agilent technologies, Santa Clara, CA, USA).

### Microarray hybridization

RNA extracted from three replicate biological samples was prepared for microarray analysis. However, technical replications were not conducted because of the high reliability and consistency of the microarray. The poplar genome array designed by Affymetrix was used. Array hybridization and washing was performed using GeneChip Hybridization, Wash and Stain Kit (Affymetrix, Santa Clara, CA, USA) in Hybridization Oven 645 (Affymetrix) and Fluidics Station 450 (Affymetrix, Santa Clara, CA, USA). All 9 gene chip procedures were performed at Shanghai Biotechnology Corp., China.

### Microarray data analysis

Slides were scanned by a GeneChip Scanner 3000 (Affymetrix) and Command Console Software 3.1 (Affymetrix). Raw data were normalized with the MAS 5.0 algorithm (Gene Spring Software 11.0; Agilent technologies). There were few degrees of freedom for the gene expression signal variance because the number of samples was lower than the number of genes [20]. Thus, the randomized variance model (RVM) F-test [21], which effectively raises the degrees of freedom in the case of smaller samples, was applied to filter the differentially expressed genes in the three treatments. After significance analysis and false discovery rate (FDR<0.05) analysis, the differentially expressed genes were selected according to the *P*-value threshold (*P*<0.05). The raw and processed data were submitted to the Gene Expression Omnibus of NCBI under the accession number GSE55216.

### Bioinformatics analysis of microarray data

After the differentially expressed genes were filtered by RVM corrective ANOVA, the genes most likely to be associated with elevated CO_2_ were further dissected by the integrated bioinformatics analysis methods cluster analysis, pathway analysis, gene ontology (GO) analysis, and signal transduction network (Signal-net) analysis (Figure S1).

#### Cluster analysis

Differentially expressed genes were further clustered using the series test of cluster algorithm of gene expression dynamics. Cluster analysis was implemented entirely in java. The cluster algorithm was used to profile the gene expression-CO_2_ concentration series and identify the most distinct clusters generating the observed series. On the basis of the change tendencies of the different signal densities of genes under different CO_2_ concentrations, a set of unique expression profiles was identified. The raw expression values were converted into log_2_ratio. Each profile contained a certain number of differentially expressed genes with similar expression patterns. The expression profiles were related to the actual or expected number of genes assigned to each profile. Significant profiles have a higher probability than expected by Fisher's exact test and the multiple comparison test [20], [22].

#### Pathway analysis

All of the differentially expressed genes contained in all significant expression profiles underwent pathway analysis. Pathway analysis was applied to the genes belonging to specific profiles to find the main biological functions of genes with the same expression trend. Analyses were based on the Kyoto encyclopedia of genes and genomes (KEGG) database using SBC Analysis System (http://sas.ebioservice.com/) at Shanghai Biotechnology Corp., China. The threshold of significance was defined as *P*<0.05.

#### Gene ontology analysis

Functional analysis was simultaneously integrated with GO classification [23]. The difference was that the differentially expressed genes were analyzed in each significant expression profile, respectively. Analyses were based on GO using DAVID bioinformatics resources (http://david.abcc.ncifcrf.gov) [24]. The differentially expressed genes were classified into several biological process categories from GO annotation for each significant expression profile. The criterion of *P*<0.05 was used to screen for significant GO terms.

#### Signal transduction network analysis

Differentially expressed genes contained in significant expression profiles were used to build gene signal transduction network (Signal-net) using Cytoscape software (version 2.8.3; www.cytoscape.org). The network was built according to the normalized signal intensity of genes. First, the Pearson's correlation was calculated for each pair of genes. Then the significantly correlated pairs were used to construct gene-gene interaction networks. Networks are stored and presented as graphs, where nodes are mainly genes and edges represent relation types between the nodes, such as activation or phosphorylation [25]. The degree is defined as the link number of one node with all of the other nodes. Genes with higher degrees occupied more important positions within the network. In addition, the properties of genes are described by Betweenness Centrality (BC) measures [25], reflecting the intermediary capacity of a node to modulate other interactions nodes [26]. Finally, the purpose of the signal transduction network analysis was to locate core key regulatory genes that had a stronger capacity to modulating adjacent genes.

### Quantitative real-time PCR validation of differentially expressed genes

The differential expression of 8 genes was confirmed by quantitative real-time PCR (qRT-PCR). The cDNA was synthesized from 2 µg of total RNA using Superscript II (Invitrogen, Carlsbad, CA, USA) with Oligo (dT) primers in 25 µL, following the manufacturer's instructions. Reactions were carried out on a 7900 HT Sequence Detection System (ABI, Carlsbad, CA, USA) with ABI Power SYBR Green PCR Master Mix and gene-specific primers. The cycling conditions consisted of an initial denaturation (95°C, 10 min), followed by 40 cycles of denaturation (95°C, 15 s), annealing, and extension (60°C, 34 s). Candidate genes were tested in triplicate wells and in three duplicate experiments. The gene expression levels were calculated relative to the expression of the poplar actin gene, a housekeeping gene [27], using the 2^-ΔΔCt^ method.

## Results

### Growth, leaf gas exchange and endogenous hormone analysis

**Figure 1:**
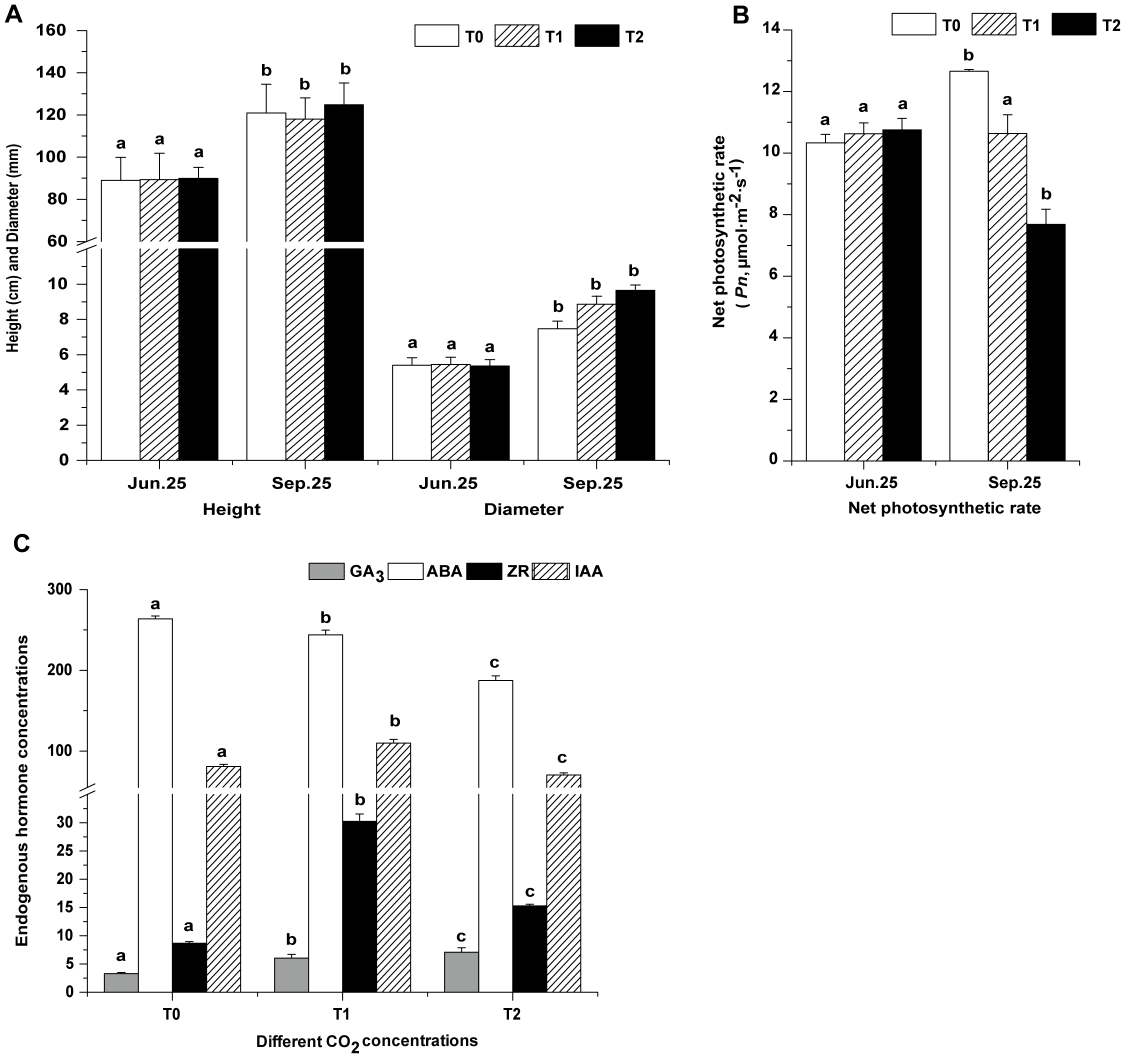
Changes in growth, net photosynthetic rate and endogenous hormones at different CO_2_ concentrations. The three CO_2_ concentrations were: T0 treatment (385 µmol·mol^−1^ CO_2_, day 25°C/night 20°C); T1 treatment (550 µmol·mol^−1^ CO_2_, day 28°C/night 23°C); and T2 treatment (720 µmol·mol^−1^ CO_2_, day 31°C/night 26°C). **A**. Growth parameters of height and diameter of trees measured on June 25 and September 25 at different CO_2_ concentrations. Bars represent SE (n = 20). **B**. Net photosynthetic rates of leaves sampled on June 25 and September 25. **C**. Levels of four endogenous hormones [gibberellic acid (GA_3_, ng/g), abscisic acid (ABA, ng/g), zeatin riboside (ZR, ng/g), and indole acetic acid (IAA, µg/g)] in leaves sampled on September 25 at different CO_2_ concentrations. The unit of four hormone concentrations is in fresh weight. Bars represent SE (n = 30 for net photosynthetic rates; n = 9 for endogenous hormone concentrations). Different letters on columns with the same pattern indicate differences at *P*<0.05 according to the LSD test.

To better understand the growth patterns of the triploid white poplar, tree height and stem diameter were measured for three months after growth initiation under different CO_2_ concentrations (Figure 1A). The diameter was significantly greater in elevated CO_2_ concentrations (increases of 18.53% for T1 and 28.96% for T2 treatment), but no significant differences were observed in height.

Unlike height and diameter, significantly decreased net photosynthetic rates were found under elevated CO_2_ concentrations on September 25 compared with the control (T0 treatment) (Figure 1B). The net photosynthetic rates of leaves were 15.93% lower for T1 and 39.24% for T2 than in plants under T0 treatment.

Compared with control, the GA_3_ concentrations increased continuously under elevated CO_2_ concentrations, consistent with the changes of diameter (Figure 1A). Similarly, the ZR concentrations under T1 and T2 treatment were always higher than that of T0 (Figure 1C). IAA concentrations were higher than the other three endogenous hormones, reaching the microgram level. The concentrations of IAA increased under T1 and decreased under T2 treatment. In contrast, the ABA concentrations decreased under elevated CO_2_ concentrations. GA3, ZR and IAA are considered to be growth promoting factors in plant development, while ABA is regarded as an inhibitory factor.

### Genes screened by ANOVA and cluster analysis

After 90 days, a total of 5,127 differentially expressed genes were identified according to RVM corrective ANOVA (*P*<0.05 and FDR <0.05) under elevated CO_2_ concentrations (Table S1). The gene expression value per treatment was the geometric mean of the robust multichip average normalized gene signals of 3 samples per CO_2_ concentration.

**Figure 2:**
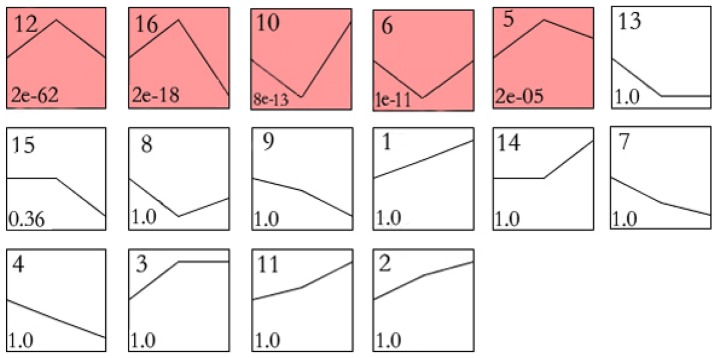
Cluster analysis of expression profiles of 5,127 differentially expressed genes. There were sixteen profiles for differentially expressed genes under elevated CO_2_ concentrations. Each box represents an expression profile. The upper number in the box is the profile number and the lower shows the *P*-value. Colored squares indicate significant profiles (*P*<0.05).

To further narrow the target genes among the 5,127 genes, sixteen expression profiles were defined by cluster analysis to summarize the expression pattern of the genes (Figure 2, Table 1). Each profile contained a cluster of genes with similar expression patterns after elevated CO_2_ concentrations treatments. As shown in Figure 2, among the 16 profiles, only 5 profiles of genes that show very significant *P*-values (*P*<0.05) (profile 12, 16, 10, 6 and 5). A total of 2,473 genes were contained in these 5 profiles (Table S2).

**Table 1:** Differentially expressed genes grouped into expression profiles by cluster analysis.

Profile number	Num. Genes Assigned*	Num. Genes Expected*	*P*-values*
Profile 1 (0,1,2)	73	127.00	1.0
Profile 2 (0,2,3)	100	172.33	1.0
Profile 3 (0,1,1)	212	304.83	1.0
Profile 4 (0,−1,−2)	72	127.00	1.0
Profile 5 (0,2,1)	440	362.67	2.24E-05
Profile 6 (0,−1,0)	498	368.33	1.13E-11
Profile 7 (0,−2,−3)	94	207.00	1.0
Profile 8 (0,−2,−1)	290	362.67	1.0
Profile 9 (0,−1,−3)	119	172.33	1.0
Profile 10 (0,−1,1)	499	362.67	8.22E-13
Profile 11 (0,1,3)	136	207.00	1.0
Profile 12 (0,1,0)	864	483.67	2.17E-62
Profile 13 (0,−1,−1)	171	403.50	1.0
Profile 14 (0,0,−1)	322	403.50	1.0
Profile 15 (0,0,−1)	311	304.83	0.37
Profile 16 (0,1,−1)	531	362.67	3.00E-18

*Num.Genes Assigned is the actual number of genes assigned to the model profile. Num.Genes Expected is the expected number of genes assigned to the model profile in a random distribution (theoretical calculation value, so not integers). *P*-values are the significance levels between actual and expected numbers of genes.

### Functional classification by Gene Ontology based on 5 significant profiles

GO analysis was conducted on the 2,473 differentially expressed genes in the 5 significant profiles (Table S3). Functional categories in biological processes from GO annotation were mainly divided into four parts: metabolic process (77.55%), response to stimulus (14.29%), cellular component organization or biogenesis (6.12%) and regulation of biological process (2.04%). According to the function enrichment values (enrichment >5 and *P*<0.05), the three most significant GO terms regulated by elevated CO_2_ were pyridine nucleotide biosynthetic process, sulfate assimilation, and carbon utilization by fixation of carbon dioxide. Among these Go terms, other than hypothetical proteins, the three genes nicotinate phosphoribosyltransferase (Nampt), nicotinamide-nucleotide adenylyltransferase (Nmnat), and quinolinate synthetase A (NadA) were found.

### Functional classification by KEGG in each significant expression profile

**Figure 3:**
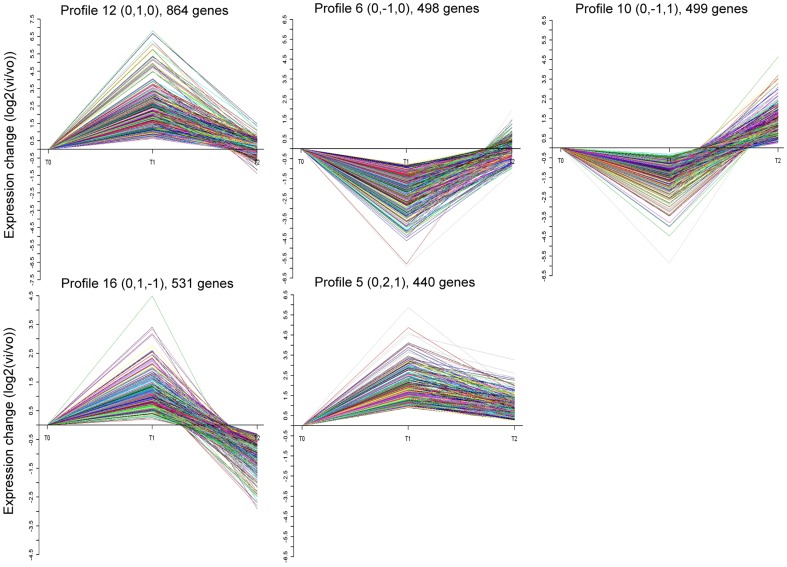
The five significant expression profiles detailed in expression graphs. Horizontal axis, CO_2_ concentration; vertical axis, expression levels of the genes after Log-normalized transformation; Vi, gene expression levels under elevated CO_2_ concentrations; V0, gene expression levels under control CO_2_ concentration.

**Figure 4:**
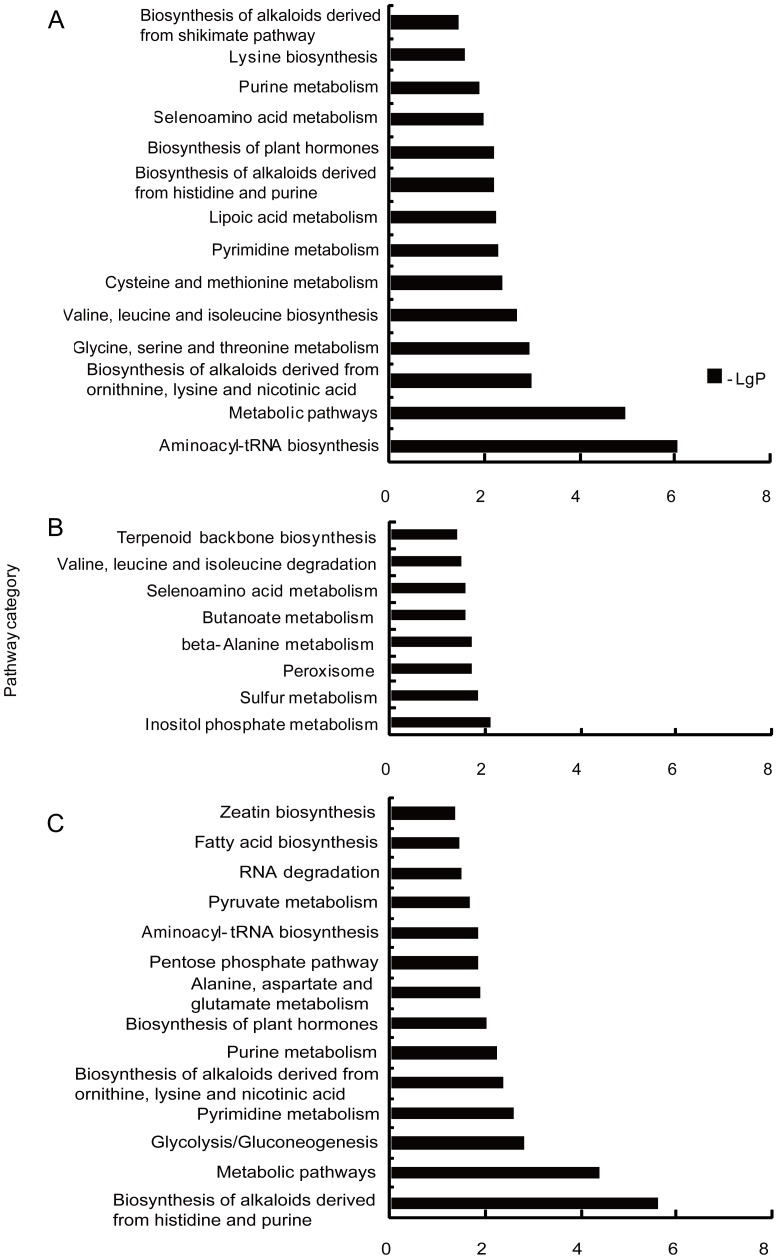
Pathway analysis based on genes in the three significant expression profiles. A, B, and C show significant pathways in profile 12, 10 and 16, respectively. Vertical axis, pathway category; horizontal axis, negative logarithm of *P*-values of pathways.

Profiles 12 and 6 showed changed expression only under T1 treatment (Figure 3), and respectively contained 864 and 498 genes. Profile 12 showed the greatest change in gene expression (increased), while profile 6 showed decreased gene expression. After functional analysis of genes by KEGG, 14 pathways in profile 12 were considered to be affected by elevated CO_2_ (Figure 4A). Only one category “carbon fixation in photosynthetic organisms” in profile 6 was found to be regulated by elevated CO_2_.

Significantly changed patterns under both T1 and T2 treatments were seen in profile 10, 16 and 5 (Figure 3). Profile 10 included 499 genes showing decreased expression under T1 and increased expression under T2 treatment. Profile 16 showed an expression tendency opposite to profile 10. The 8 and 14 pathways were markedly affected by elevated CO_2_ in profile 10 (Figure 4B) and 16 (Figure 4C), respectively. Unlike the other expression pattern, profile 5 contained 440 genes whose expression increased under both elevated CO_2_ concentrations treatments. Most of these genes are associated with non-homologous end-joining and methane metabolism pathways.

### Signal transduction network analysis from the five significant profiles

**Figure 5:**
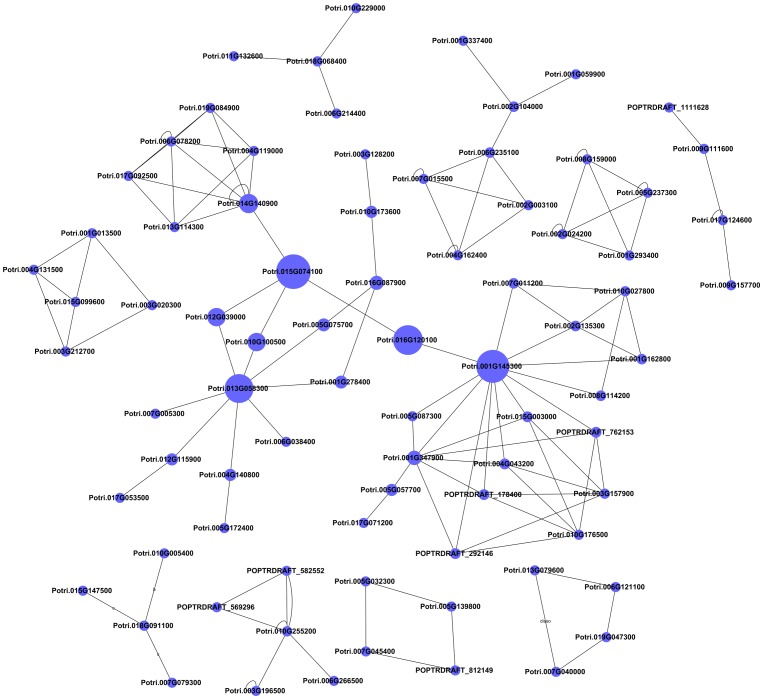
Signal transduction network of differentially expressed genes under different CO_2_ concentrations. Cycle nodes represent genes, the sizes of nodes represent the power of the interrelation among the nodes, and the edges between two nodes represent interactions between genes. Details of genes that mapped to each cycle node are listed in Table S4.

Among the five significant profiles, 2,473 differentially expressed genes were analyzed using the signal transduction network with the BC algorithm to determine which genes play an important role under elevated CO_2_ concentrations (Figure 5). In the network, cycle nodes represent genes, and edges between two nodes represent interactions between genes, which are expressed by degree, where indegree represent the number of source genes of a gene and outdegree represent the number of target genes of a gene [28]. Degree measures how correlated a gene is with all other network genes.

Combined with the interactions among genes, the BC values of each gene were obtained (Table S4). The characteristics of genes described by BC values reflect the importance of a gene related to other genes [29]. The BC values showed whether one gene regulates and controls other genes, or the interaction with other genes in the network. The higher the BC value, the more modulation there is between genes. The genes with high BC values provided key genes with a strong capacity to modulate adjacent genes under elevated CO_2_ concentrations.

Nine genes with higher BC values under elevated CO_2_ concentrations were validated by Signal-net analysis (Table 2). Core genes like pyruvate kinase (PK) and aldehyde dehydrogenase (NAD+) (ALDH) appeared at the center of the network and both had high BC and degree values. They were both transcriptionally up-regulated under T1 treatment and not significantly changed under T2 treatment. However, the expression abundance of the gene asparagine synthase (glutamine-hydrolyzing) (Potri.001G278400) was undetectable by qRT-PCR (the primers used are listed in Table S5). Only the expression of 8 genes was quantified by qRT-PCR (Table 3). A positive correlation (r = 0.743, *P* <0.01) of transcription trends between microarray and qRT-PCR was obtained. The 8 genes were ALDH, PK, pyruvate decarboxylase (PDC), glutamate dehydrogenase (NAD(P)+) (GDH), acetate-CoA ligase (ACAS), adenylosuccinate synthase (AdSS), asparagine synthase (glutamine-hydrolyzing) (AS), and nitrite reductase (NiR), which changed significantly under elevated CO_2_ concentrations (Tables 2 and 3).

**Table 2:** Nine key genes confirmed by Signal-net analysis.

Gene symbol	Gene description	Gene abbreviations	Betweenness Centrality*	Degree*
Potri.015G074100	aldehyde dehydrogenase (NAD+)	ALDH	0.998876	4
Potri.001G145300	pyruvate kinase	PK	0.932210	12
Potri.016G120100	pyruvate decarboxylase	PDC	0.802247	2
Potri.013G058300	glutamate dehydrogenase (NAD(P)+)	GDH	0.766292	8
Potri.014G140900	acetate-CoA ligase	ACAS	0.372846	7
Potri.016G087900	adenylosuccinate synthase	AdSS	0.162921	3
Potri.005G075700	asparagine synthase (glutamine-hydrolyzing)	AS	0.114607	2
Potri.001G278400	asparagine synthase (glutamine-hydrolyzing)	AS	0.114607	2
Potri.004G140800	nitrite reductase	NiR	0.083146	2

*Genes are named according to the newest version of *Populus trichocarpa* 3.0. Betweenness Centrality measures how correlated a gene is with all other network genes. Degree describes the number of single genes that regulate other genes and represents the size of the cycle node.

**Table 3:** Expression levels of 8 genes quantified by qRT-PCR.

Gene symbol	Sample number	2^-ΔΔCt^ (T1/T0)	2^-ΔΔCt^ (T2/T0)
Potri.015G074100	9	1.740±0.103*	0.607±0.115*#
Potri.001G145300	9	1.511±0.310*	0.945±0.186#
Potri.016G120100	9	1.408±0.367	0.448±0.081#
Potri.013G058300	9	0.523±0.216*	0.909±0.367
Potri.014G140900	9	1.062±0.137	0.757±0.079*#
Potri.016G087900	9	1.420±0.094*	0.498±0.064*#
Potri.005G075700	9	1.750±0.189*	0.975±0.047#
Potri.004G140800	9	2.382±0.731	0.509±0.160#

*P<0.05 compared with T0; #*P*<0.05 compared with T1.

## Discussion

In terms of physiological processes, the growth parameter of diameter was significantly increased and net photosynthetic rate was decreased under elevated CO_2_ concentrations. Simultaneously, the changes in concentration of the four endogenous hormones (GA3, ZR, IAA, and ABA) appeared to actively promote plant development. These changes in physiological parameters prompted us to study the molecular processes at the transcriptome level. In this study, we focused on gene expression in the triploid white poplar leaf under elevated CO_2_ concentrations using gene chips, in order to confirm the key genes affected.

Firstly, after the selection of 5,127 differentially expressed genes under elevated CO_2_ concentrations, a set of unique and representative expression profiles was identified. Significant profiles indicate that common functions attributable to the co-expressed genes [20]. Such functions mainly indicate the biological characteristics [30]. With this method, we explicitly considered the dynamic nature of gene expression profiles during clustering and confirmed a number of clear clusters [31].

Second, after filtering the differentially expressed genes by significant expression profiles, functional annotation based on GO analysis showed that several genes functioned in metabolic process (77.55%) and response to stimulus of external environment (14.29%), including response to light stimulus, radiation, abiotic stimulus and stress, the latter containing cellular response to stress, base-excision repair, DNA repair, and response to DNA damage stimulus. It is worthy of note that 31 genes participating in the function of “response to stimulus of external environment” contributed to the delivery of important signal molecules responding to elevated CO_2_. One gene for a photoreceptor-interacting protein was found, which showed decreased expression under T1 and increased expression under T2 treatment. In short, metabolism-related genes displayed considerable responses to elevated CO_2_ [5], [6].

In addition, a similar phenomenon was found in KEGG analysis. KEGG annotation showed that most of pathways were related to metabolism, including the metabolism of amino acids (glycine, serine and threonine metabolism), carbohydrates (glycolysis/gluconeogenesis), nucleotides (pyrimidine metabolism), cofactors and vitamins (lipoic acid metabolism), and energy (carbon fixation in photosynthetic organisms); these were abundant in all significant pathways. Some have already been reported to participate in responses to elevated CO_2_. For example, studies have shown that elevated CO_2_ induced an increase in transcripts associated with glycolysis in soybean, rice and aspen [5], [9], [13], [16], [32]. The pathway analysis identified differentially expressed genes involved in the biosynthesis of plant hormones pathway [5] and zeatin (Figure 4). Meanwhile, we found changes in endogenous hormone concentrations.

Furthermore, at the same time, molecular network maps were constructed using differentially expressed genes in significant gene expression profiles. The Signal-net analysis method was used to screen for the source gene or target gene of some gene in whole KEGG-pathway database [28]. The BC values and degrees of genes are the key attributes of a network; they show the tendency of genes to interconnect with others and were used to seek out major target genes. These genes were located at the core of the network after increased CO_2_ treatment.

Finally, after a series of biological and bioinformatics analyses, 8 genes were identified in the network and confirmed by qRT-PCR. All 8 genes (ALDH, PK, PDC, GDH, ACAS, AdSS, AS, and NiR) were significantly altered by elevated CO_2_ concentrations. Thus, these methods are effective for analyzing the data from gene chips to gain valuable information [20], [31]. It will be important to explore the variations in poplar to identify genes that are ‘pre-adapted’ to future conditions of elevated CO_2_ in global climate change.

Among the 8 genes, PK had both higher BC values and degree; it is a key regulator of the step between carbon metabolism and protein synthesis and a number of transcription factors [11]. The transcript abundance of PK is also significantly altered in soybean in an elevated CO_2_ concentration (550 µmol·mol^−1^) compared to the ambient CO_2_ concentration [9]. However, contradictory research results have been found in other studies. Decreased gene expression of PK under elevated CO_2_ has been reported [11], [32] whereas increased PK transcripts was reported by Fukayama et al. [6]. In addition, PK is an important enzyme in the glycolytic pathway that also functions in providing carbon skeleton for fatty acid biosynthesis in plants [33]. Ainsworth et al. [5] concluded that the transcript levels of genes associated with fatty acid biosynthesis was increased in soybean under elevated CO_2_ (550 µmol·mol^−1^). All these results suggest that this is an interesting and important gene for further analysis of responses to future rising CO_2_. Another key gene in this study was ACAS, which is produced needed for fatty acid synthesis, but under normal conditions the gene is inactive; specific factors activate its transcription when necessary [34].

Another key gene with higher BC values and degree was glycolysis related ALDH. The importance of ALDH genes in the stress response has been investigated by analyzing transgenic *Arabidospsis thaliana* [35]. Kontunen-Soppela et al. [36] and Leakey et al. [9] found that, under elevated CO_2_ concentration (550 µmol·mol^−1^), the gene expression of ALDH was changed in paper birth and soybean, respectively. Moreover, the progressive inhibitors of the activity of enzymes of nitrogen metabolism, NiR and GDH, were two other key genes in our study. Some researchers have reported that NiR is up-regulated in soybean [37] and GDH is changed in paper birth [36] under elevated CO_2_. In addition, the key gene AS plays an important role in amino-acid biosynthesis. It functions in various metabolic processes, such as cellular amino acid, organic acid, carboxylic acid, and amine biosynthetic process (Table S3). It is down-regulated in early-season *P. tremuloides* leaves under elevated CO_2_ (560 µmol·mol^−1^) concentration [16]. In general, these genes with important functions were clearly changed in plants under increased CO_2_ concentrations. As a result, they may be potential target genes for further research. In future study, our ultimate goal is to confirm the functions of key genes in poplar affected by elevated CO_2_.

## Conclusions

The changes in physiological parameters in response to elevated CO_2_ concentrations encouraged us to study the molecular processes at the transcriptome level using microarray. To understand the key genes related to elevated CO_2_, as well as the pathways and biological processed using the gene chip data, 8 significantly expressed genes were selected and confirmed by integrated bioinformatics methods. The 8 genes were ALDH, PK, PDC, GDH, ACAS, AdSS, AS, and NiR, dedicated to metabolism and responses to stimulus of external environment. These genes have crucial effects in the network and strong correlations with elevated CO_2_ concentration treatments, and are worthy of further exploration. This study provides several target genes that could be used in initial steps for better understanding the molecular mechanisms of plant acclimation and evolution in future rising CO_2_ concentrations.

## Supporting Information

Figure S1 **Flowchart of bioinformatics analysis for identifying key genes responding to elevated CO_2_ concentrations.**
(TIF)

Table S1 **List of 5,127 significant differentially expressed genes.**
(XLS)

Table S2 **The five significant expression profiles identified by cluster analysis.**
(XLS)

Table S3 **Significant functional annotation and categories of differentially expressed genes based on biological processes of Gene Ontology.**
(XLS)

Table S4 **Interactions between genes and genes identified by Signal-net analysis under different CO_2_ concentrations.**
(XLS)

Table S5 **Primers of 9 selected genes and 1 housekeeping gene used for qRT-PCR.**
(DOC)
