# Supplementary figures and images for: Genes Responsive to Elevated CO2 Concentrations in Triploid White Poplar and Integrated Gene Network Analysis
Source: PLoS One. 2014 May 21;9(5):e98300. doi: 10.1371/journal.pone.0098300 (PMC4029852; doi:10.1371/journal.pone.0098300)

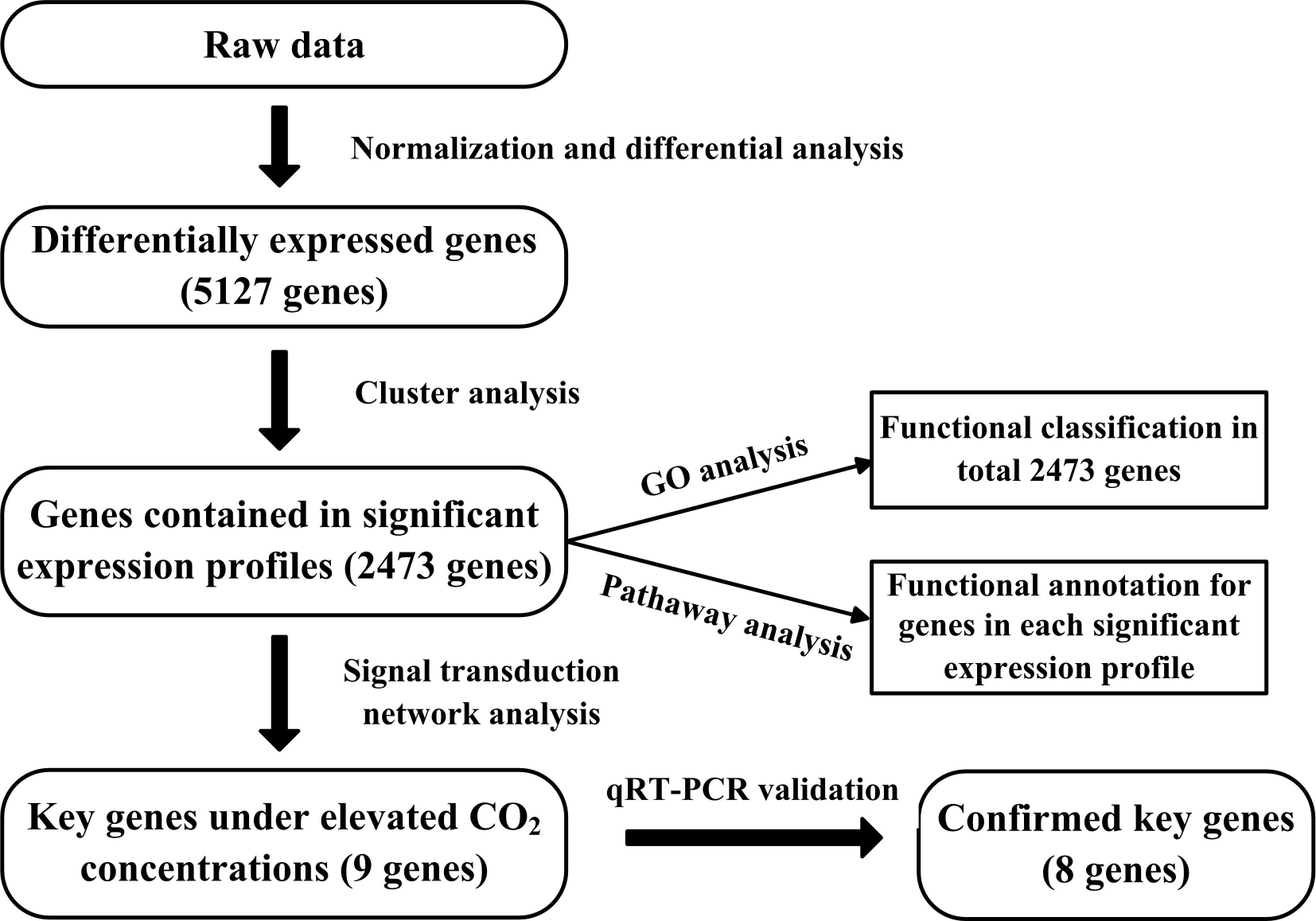

Supplement: Figure S1 — Flowchart of bioinformatics analysis for identifying key genes responding to elevated CO2 concentrations. (TIF) [file pone.0098300.s001.tif]
