# Supplementary material for: Genes Responsive to Elevated CO2 Concentrations in Triploid White Poplar and Integrated Gene Network Analysis
Source: PLoS One. 2014 May 21;9(5):e98300. doi: 10.1371/journal.pone.0098300 (PMC4029852; doi:10.1371/journal.pone.0098300)
Supplement: Table S5 — Primers of 9 selected genes and 1 housekeeping gene used for qRT-PCR. (DOC) [file pone.0098300.s006.doc]

**Table S5. Primers of 9 selected genes and 1 housekeeping gene used for qRT-PCR.**

| Probe set ID | Primer Name | Sequence 5’-3’ |
| --- | --- | --- |
| POPTRDRAFT_658391 | p-actin-F | TCGCAGATTACAACAGTACAGCA |
|  | p-actin-R | TGGCAACCTCTTCATCCAGA |
| POPTRDRAFT_666446 | P-NAD+-F | ACGTTTATTTCGTTACTATGCTGG |
|  | P-NAD+-R | GAAAGTTCCAAGGGATTATCTGTC |
| POPTRDRAFT_1109792 | P-pyruvate kinase-F | GCTATACATTTAAGTCATTTTCGGC |
|  | P-pyruvate kinase-R | CCAGCCTCTGCTTTATCCTTTC |
| POPTRDRAFT_835585 | P-p.decarboxylase-F | AATAGCCGTCCTCCAAACCCT |
|  | P-p.decarboxylase-R | GTAACTAAGAAGCATAGCATCATCACATA |
| POPTRDRAFT_571209 | P-NAD(P)+-F | GGCAACCAACAGGAACTTCAAG |
|  | P-NAD(P)+-R | AAGTGCCATCATCTTTGGGTATG |
| POPTRDRAFT_572831 | P-CoA ligase-F | AACATTCAAGTATGCTTTTGATTACAA |
|  | P-CoA ligase-R | GGTGATCCAACCACAATCAGC |
| POPTRDRAFT_825248 | P-a.synthase-F | TTGGAGCCGTTCATTACTGATAC |
|  | P-a.synthase-R | CTAACATGGTAGCTTGACCACCTT |
| POPTRDRAFT_207428 | P-asparagine-F | GGTTGAAGCATAGAGGACCAGATT |
|  | P-asparagine-R | CAGGGTCTACAATAGCCAAACG |
| POPTRDRAFT_829702 | P-glutamine-h-F | CAAAGTTAGCTTCTGCTGGAGGA |
|  | P-glutamine-h-R | ACAACAAGGTGCCAACACTACTACA |
| POPTRDRAFT_837131 | P-n.reductase-F | GGACTTGGTGCCTTTGGTTG |
|  | P-n.reductase-R | TAATCATCCACCTCTTCCCTCTC |
